# Single-cell and spatial dissection of IGF2BP3-driven endothelial reprogramming underlying microvascular invasion in hepatocellular carcinoma

**DOI:** 10.3389/fphar.2026.1781301

**Published:** 2026-04-01

**Authors:** Xinglong Zhang, Huarong Fu, Jun Lu

**Affiliations:** Department of Oncology, Anhui Zhongke Gengjiu Hospital, Hefei, Anhui, China

**Keywords:** drug targets, endothelial heterogeneity, hepatocellular carcinoma, IGF2BP3, microvascular invasion

## Abstract

**Background:**

Microvascular invasion (MVI) is a critical determinant of early recurrence and poor prognosis in hepatocellular carcinoma (HCC). The cellular and molecular mechanisms driving MVI, particularly the role of endothelial heterogeneity, remain incompletely understood.

**Methods:**

We integrated single-cell and spatial transcriptomics from multiple HCC cohorts to identify MVI-associated endothelial subpopulations. GeneNMF and hdWGCNA were applied to define stable gene modules, while CellChat and NicheNet analyzed intercellular signaling. Prognostic relevance was assessed using machine learning–based risk models across TCGA-LIHC and external datasets. Functional assays including EdU proliferation, tube formation, and Western blot were conducted to validate the role of IGF2BP3 in tumor-associated endothelial cells.

**Results:**

We identified a distinct MVI-associated endothelial subpopulation (MVI Endo) enriched in pro-angiogenic, EMT-like, and TGF-β-responsive pathways, predominantly localized near tumor vasculature. MVI Endo engaged in robust VEGF, ANGPT2, and TGF-β signaling with tumor and stromal cells. Machine learning–derived prognostic models highlighted IGF2BP3 as a central regulator, and functional assays demonstrated that IGF2BP3 enhances endothelial proliferation and tube formation via upregulation of VEGF-A and ANGPT2, supporting its involvement in the vascular phenotype associated with MVI.

**Conclusion:**

This study characterizes the endothelial landscape associated with MVI, implicates IGF2BP3 as a critical regulator of vascular reprogramming, and provides a foundation for precision vascular-targeted therapies in HCC.

## Introduction

Hepatocellular carcinoma (HCC) is a highly heterogeneous malignant tumor with continuously increasing incidence and mortality worldwide. Despite advances in surgical techniques and systemic therapeutic strategies, long-term survival of patients with HCC remains substantially compromised, with early postoperative recurrence representing a major contributor to unfavorable outcomes (Sangro et al., 2021). Among pathological features, microvascular invasion (MVI) is widely recognized as one of the most aggressive indicators and a powerful predictor of poor prognosis, reflecting the ability of tumor cells to infiltrate vascular structures and disseminate at an early stage. However, the cellular ecological characteristics and molecular mechanisms underlying MVI have not been fully elucidated. In particular, the limited resolution of conventional bulk sequencing approaches restricts the identification of key cellular sources and signaling networks involved in MVI (Llovet et al., 2022; Forner et al., 2018). The rapid development of single-cell sequencing and spatial transcriptomic technologies has enabled unprecedented resolution of the tumor microenvironment, creating new opportunities to uncover MVI-specific cellular states and regulatory pathways.

Recent multi-omics studies have demonstrated that the occurrence of MVI is closely associated with endothelial reprogramming, extracellular matrix remodeling, epithelial–mesenchymal transition (EMT), and activation of multiple pro-angiogenic signaling pathways. Tumor-associated endothelial cells (TAECs) frequently exhibit activation of VEGF, ANGPT2, and TGF-β signaling, which compromises vascular integrity and increases susceptibility to tumor cell penetration (Yao et al., 2025). In parallel, tumor cells enhance their invasive capacity and reshape the local vascular niche through upregulation of the RNA-binding protein IGF2BP3 and other pro-migratory factors. Notably, IGF2BP3 has been identified as an m^6^A reader that stabilizes NRF2 mRNA, thereby enhancing antioxidant capacity and cellular tolerance, which contributes to sorafenib resistance in HCC (Lu et al., 2024). These findings indicate that IGF2BP3 plays a critical role in regulating malignant phenotypes and therapeutic responses. Emerging evidence further suggests that IGF2BP3 stabilizes transcripts of multiple pro-angiogenic molecules, including VEGFA and ANGPT2, thereby promoting endothelial cell proliferation, tube formation, and vascular permeability, ultimately creating a permissive microenvironment for microvascular invasion (Xu et al., 2025). Consequently, identifying endothelial subpopulations with MVI-specific molecular features and their key regulatory factors is essential for a mechanistic understanding of MVI.

Distinct from prior single-cell studies that largely focused on the immune landscape or tumor heterogeneity of MVI, the present study aimed to systematically dissect the endothelial-specific reprogramming underlying MVI. By integrating single-cell transcriptomic and spatial transcriptomics, we not only identified a specialized MVI-associated endothelial subpopulation (MVI Endo) characterized by pro-angiogenic, EMT-like, and TGF-β–responsive features but also visually mapped its specific enrichment at the invasive tumor edge. Furthermore, we moved beyond descriptive analysis to mechanistically validate IGF2BP3 as a key regulator of this vascular phenotype and translated these findings into a robust machine learning–based prognostic model. This multi-modal approach establishes a comprehensive spatial-molecular atlas of endothelial heterogeneity in MVI, offering novel mechanistic insights and precision therapeutic targets.

## Methods

### Single-cell quality control and annotation

Single-cell RNA-seq data from GSE242889 (Li et al., 2024) were obtained from the Gene Expression Omnibus (GEO) and integrated for downstream analyses. Doublets were identified and removed with DoubletFinder, and ambient RNA contamination was corrected with decontX. Cells with >300 detected genes and mitochondrial gene content <20% were retained. Batch effects were corrected with the harmony algorithm. Marker genes for each cluster were identified with FindAllMarkers, and cell types were annotated and visualized with reference to prior reports (Li et al., 2024) and CellMarker 2.0 (Hu et al., 2023). Bulk transcriptomic data with survival information for LIHC were retrieved from the University of California Santa Cruz (UCSC) database and applied to model validation through **HCCDB18**. In addition, E-TABM-36, GSE10141, GSE144269, GSE14520, GSE27150, and GSE76427 were used as external cohorts for prognostic validation. Differentially expressed genes between tumor and normal tissues were identified with limma (Ritchie et al., 2015), with a threshold of *p value* < 0.05.

### GeneNMF

Non-negative matrix factorization (NMF) is a high-dimensional data analysis approach that extracts sparse and interpretable features from non-negative matrices. It is well suited to single-cell transcriptomic data, enabling reduction of large gene-by-cell matrices into a limited number of biologically interpretable gene programs. GeneNMF (Barkley et al., 2022; Gavish et al., 2023) implements NMF for single-cell omics and can be applied directly to Seurat objects to reduce dimensionality and identify robust gene programs across multiple samples. GeneNMF was applied to endothelial cell subsets to identify consensus NMF programs shared across samples, thereby defining stable endothelial subpopulations for subsequent analyses. Prognostic analyses were then conducted on transcriptomic profiles derived from markers of these robust subpopulations to identify key prognostically relevant endothelial states.

### hdWGCNA

hdWGCNA (high-dimensional weighted gene co-expression network analysis) (Morabito et al., 2021) was applied to perform WGCNA in high-dimensional transcriptomic data, including single-cell RNA-seq and spatial transcriptomics. This framework enables construction of modular co-expression networks across multiple cellular and spatial scales and identification of highly interconnected gene modules with biological context. By comparing the distribution of MVI-related signals within endothelial cells, hdWGCNA was used to identify the most relevant MVI-associated co-expression modules.

### Single-cell metabolic analysis

To systematically quantify the metabolic activity of endothelial subpopulations, we employed the scMetabolism package (v0.2.1) in R. This algorithm evaluates metabolic pathway activities at the single-cell level based on a curated reference gene set covering 85 metabolic pathways from the KEGG and REACTOME databases. The “VISION” scoring method was utilized to calculate metabolic scores for each individual cell. The average metabolic scores were then compared between the MVI Endo subpopulation and other endothelial clusters to identify specific metabolic reprogramming features.

### SCENIC analysis

SCENIC (Aibar et al., 2017) was applied to characterize transcriptional regulatory networks and cell states at single-cell resolution. Transcription factor (TF) regulatory networks were inferred within each subpopulation with **py**SCENIC under Python 3.10. Enrichment analysis was performed with the RcisTarget database to identify regulatory relationships between transcription start sites (TSSs) and target genes.

### Cell–cell communication and pseudotime analysis

Cell–cell communication was analyzed with CellChat (Jin et al., 2021) and the built-in CellChatDB.human reference to infer intercellular interactions across 32 signaling pathways. NicheNet (Browaeys et al., 2020) was subsequently applied to validate ligand–receptor interactions and identify key signaling routes. Pseudotime trajectories were reconstructed with Monocle2 (Bray et al., 2016) to infer directional transitions and ordering of cellular states during development or differentiation.

## Spatial transcriptomics

Paired spatial transcriptomic sections HCC-2T, HCC-2P, and HCC-2L were analyzed (Wu et al., 2021). Tumor regions were delineated with the Cottrazm package to resolve boundary areas between malignant and non-malignant regions. Each spatial spot was annotated according to cell-type composition, and spots were labeled by dominant cell types. Spearman correlation analysis was performed to assess associations between cell-type proportions and gene-set AUC scores across all spots, with visualization through linkET. Spatial deconvolution was conducted with CellTrek, and spatial co-localization analysis was performed with CARD (Ma and Zhou, 2022).

### Construction and validation of prognostic models

Based on selected prognostic genes, a consensus integrative regularized learning strategy was implemented to achieve high accuracy and stability. Ten machine-learning algorithms and 101 algorithmic combinations were evaluated, including random survival forest (RSF), elastic net (Enet), Lasso, Ridge, stepwise Cox regression, CoxBoost, Cox partial least squares regression (plsRcox), supervised principal components (SuperPC), generalized boosted regression modeling (GBM), and survival support vector machines (survival-SVM). The workflow comprised: (a) identification of differentially expressed genes between high- and low-scoring endothelial cells, followed by intersection with differentially expressed genes from the Apelin/APJ gene set to obtain key candidates; (b) fitting of 101 algorithmic combinations within a leave-one-out cross-validation (LOOCV) framework in the TCGA-LIHC cohort; (c) validation in independent datasets; and (d) calculation of the Harrell concordance index (C-index) across all validation cohorts for each model, with the model showing the highest mean C-index selected as optimal. Patients were stratified by the optimal cutoff of model scores, and survival analyses were conducted with the **survival** package. For multi-cohort integration, meta-analysis of univariate Cox results was performed with the Meta package based on the inverse-variance method, with log hazard ratio (HR) as the primary metric. HR values < 1 indicated tumor-suppressive associations, whereas HR values > 1 indicated tumor-promoting associations.

### Prognostic model–associated immune infiltration and immunotherapy prediction

The **ESTIMATE** algorithm (Yoshihara et al., 2013) was applied to expression data to infer stromal and immune cell scores in malignant tumor tissues. Immune cell composition differences between high- and low-risk groups were quantified with CIBERSORT (Newman et al., 2015), EPIC(Racle et al., 2017), MCPCounter (Becht et al., 2016), TIMER (Li et al., 2016), and xCell (Aran et al., 2017) implemented in the IOBR package (Zeng et al., 2021). The subclass mapping (submap) algorithm (Hoshida et al., 2007) was used to predict the likelihood of response to immunotherapy based on the prognostic model, while the TIP algorithm was applied to estimate functional differences of T cells between groups. Gene set enrichment analysis (GSEA) was conducted with the GseaVis package. In addition, correlations between the prognostic model and key estimated pathways were calculated by **z-score**–based scoring derived from model genes (Lee et al., 2008).

### Cell culture and transfection

Human umbilical vein endothelial cells (HUVECs) and human hepatocellular carcinoma–derived endothelial cells (ECDHCC-1) were obtained from the Typical Culture Reserve Center of China (Shanghai, China). Cells were maintained in Dulbecco modified Eagle medium (DMEM; Gibco, United States) supplemented with 10% fetal bovine serum (Gibco, United States) and 1% penicillin–streptomycin (Gibco, United States) at 37 °C in a humidified atmosphere containing 5% CO_2_. The target sequences for small interfering RNA and overexpression constructs were as follows (Table 1).

**TABLE 1:** The target sequences for small interfering RNA and overexpression constructs.

Gene	Sense/Forward (5′–3′)	Antisense/Reverse (5′–3′)
si-IGF2BP3	CCU​UGA​AAG​UAG​CCU​AUA​UTT	AUA​UAG​GCU​ACU​UUC​AAG​GTT
OE-IGF2BP3	GCG​GAA​TTC​GCC​ACC​ATG​AAC​AAA​CTG​TAT​ATC​GGA​AAC	GTT​TCC​GAT​ATA​CAG​TTT​GTT​CAT​GGT​GGC​GAA​TTC​CGC

#### EdU cell proliferation assay

Cell proliferation of ECDHCC-1 cells was assessed with the BeyoClick™ EdU Cell Proliferation Kit containing Alexa Fluor 594 (Beyotime, Shanghai, China). Cells transfected with si-IGF2BP3, oe-IGF2BP3, or corresponding controls (si-NC, oe-NC) were seeded in 24-well plates. When cell confluence reached approximately 70%, EdU working solution was added and cells were incubated for 2 h. Cells were then fixed with 4% paraformaldehyde, permeabilized with 0.5% Triton X-100, and processed according to the manufacturer’s protocol. Nuclei were counterstained with DAPI, and images were acquired with an inverted fluorescence microscope (Olympus, Japan). The proportion of EdU-positive cells was quantified to evaluate proliferative activity.

### *In Vitro* angiogenesis (tube formation) assay

*In vitro* angiogenesis was evaluated with Matrigel matrix (Corning, United States). Pre-chilled Matrigel (40 µL per well) was added to 96-well plates and allowed to polymerize at 37 °C for 30 min. ECDHCC-1 cells (1.2 × 10^4^ per well) from the si-NC, si-IGF2BP3, oe-NC, and oe-IGF2BP3 groups were seeded onto the Matrigel and incubated at 37 °C. Tube-like structures were imaged with an inverted microscope (Leica, Germany) at 6 h post-seeding. The number of branch points was quantified with ImageJ to assess tube-forming capacity.

### Western blot analysis

Total protein was extracted from ECDHCC-1 cells using RIPA lysis buffer (Beyotime, China) supplemented with protease and phosphatase inhibitors. Protein concentrations were determined by the BCA method (Beyotime, China). Equal amounts of protein (20 µg) were resolved by 10% SDS-PAGE and transferred onto 0.45 µm PVDF membranes (Millipore, United States). Membranes were blocked with 5% non-fat milk for 2 h at room temperature and incubated overnight at 4 °C with primary antibodies. The primary antibodies used were: anti-VEGF-A (1:1000, Proteintech, Cat. 81323-2-RR), anti-ANGPT2 (1:1000, Proteintech, Cat. 83816-1-RR), and anti-GAPDH (1:5000, Proteintech, Cat. 60004-1-Ig) as an internal control. After washing with TBST, membranes were incubated with HRP-conjugated secondary antibodies (1:5000, Proteintech) for 1 h at room temperature. Protein bands were visualized using an enhanced chemiluminescence (ECL) kit (Tanon, China) and imaged with a Tanon 4,600 system. Band intensities were quantified using ImageJ software (NIH, United States).

### Statistical analysis

All statistical analyses were performed using R software (version 4.2.0) and GraphPad Prism 9.0 (GraphPad Software, United States). For bioinformatic analyses, the Wilcoxon rank-sum test was used to compare continuous variables (e.g., gene expression, pathway scores) between two groups, and the Kruskal–Wallis test was used for comparisons among multiple groups. Correlations were assessed using Spearman’s or Pearson’s correlation coefficients. For experimental assays (EdU, tube formation, Western blot), data are presented as mean ± standard deviation (SD) from at least three independent experiments. Differences between two groups were analyzed using the two-tailed Student’s t-test, while comparisons among multiple groups were analyzed using one-way ANOVA followed by Tukey’s *post hoc* test. Survival curves were generated using the Kaplan-Meier method and compared using the log-rank test. A p-value <0.05 was considered statistically significant (*p < 0.05, **p < 0.01, ***p < 0.001).

## Results

### Single-cell landscape

**FIGURE 1:**
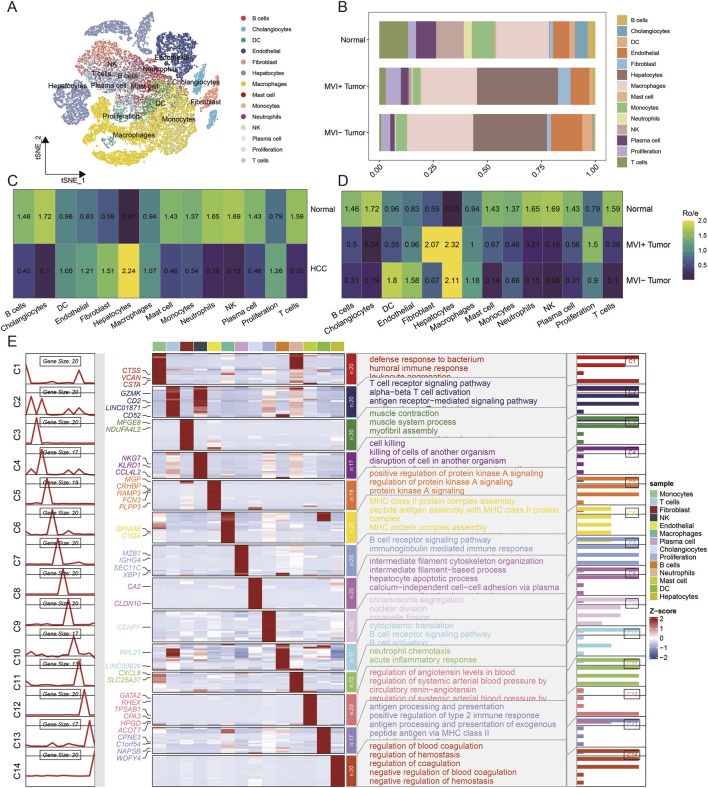
Single-cell landscape. **(A)** t-SNE visualization of single-cell clusters; **(B)** bar plot of cell proportions; **(C)** Ro/e heatmap of cell proportions between tumor and normal tissues; **(D)** Ro/e heatmap of cell proportions among MVI-positive, MVI-negative, and normal tissues; **(E)** heatmap of cell markers and functional enrichment.

Annotation of the single-cell transcriptomic data identified 14 major cell populations, including T cells, natural killer (NK) cells, endothelial cells, fibroblasts, tumor cells, monocytes, macrophages, neutrophils, B cells, mast cells, plasma cells, dendritic cells, proliferating cells, and cholangiocytes (Figure 1A). The top three marker genes for each population highlighted key differentially expressed genes across clusters (Figure 1B). Cell proportion analysis revealed distinct differences in the relative abundance of these populations (Figure 1B). Tumor cells were exclusively enriched in tumor samples (Figure 1C), whereas immune cells were highly represented in normal tissues and showed reduced enrichment in tumor tissues (Figure 1D). Functional enrichment analysis of marker genes for each population recapitulated their expected biological functions, supporting the accuracy of cell-type annotation (Figure 1E).

## Identification of key MVI-associated endothelial subpopulations

**FIGURE 2:**
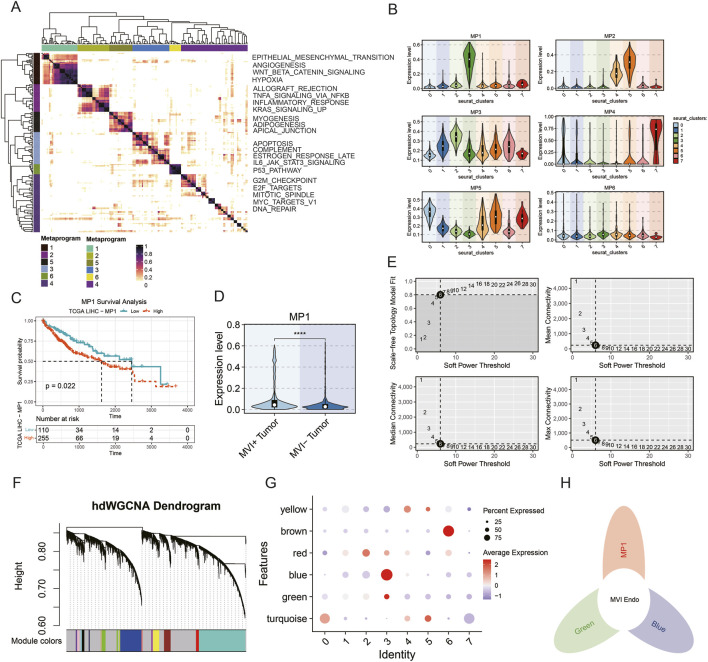
Identification of key MVI-associated endothelial subpopulations. **(A)** Heatmap of GeneNMF modules and functional enrichment; **(B)** violin plots showing module enrichment across clusters; **(C)** survival curve associated with the M1 module; **(D)** differential expression of the M1 module; **(E)** soft-threshold selection for hdWGCNA; **(F)** dendrogram of gene clustering; **(G)** dot plot of module enrichment across clusters; **(H)** Venn diagram of core MVI Endo genes.

Further analysis focused on endothelial cell subsets. GeneNMF partitioned endothelial cells into six distinct modules with divergent functional characteristics. Among these, the M1 module showed elevated expression of pathways related to angiogenesis, epithelial–mesenchymal transition (EMT), and WNT signaling, which are associated with tumor progression and MVI, whereas the remaining modules were enriched for cell-cycle regulation, cell adhesion, and immune-related pathways (Figure 2A). Module distribution analysis demonstrated that the M1 module was predominantly enriched in cluster 3, indicating a distinct endothelial subpopulation (Figure 2B). Deconvolution based on module genes revealed that the M1-associated subpopulation was linked to poorer prognosis (Figure 2C), and that the M1 module was significantly enriched in MVI-positive endothelial cells (Figure 2D). To further refine key endothelial subpopulations, hdWGCNA was applied. After soft-threshold selection and hierarchical clustering (Figures 2E,F), the blue and green modules were found to be significantly enriched in cluster 3 (Figure 2G). Based on median dichotomization of M1 module gene expression, cells with high M1 scores within cluster 3 were defined as the MVI Endo subpopulation. The intersection of genes from the M1 module and the blue and green hdWGCNA modules was therefore defined as the core gene set of MVI Endo (Figure 2H).

### Pseudotime and functional characterization of the MVI endo subpopulation

**FIGURE 3:**
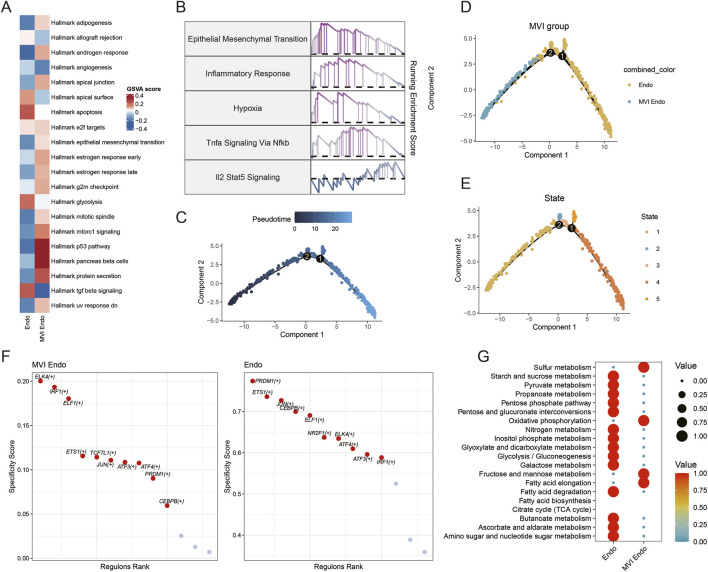
Pseudotime and functional analysis of the MVI Endo subpopulation. **(A)** Functional enrichment analysis; **(B)** GSEA results; **(C–E)** pseudotime trajectory analysis; **(F)** SCENIC-based regulatory network analysis; **(G)** metabolic activity analysis.

Functional enrichment analysis comparing MVI Endo with conventional endothelial cells showed that MVI Endo exhibited higher activity in adipogenesis, cell adhesion, EMT, and p53-related pathways (Figure 3A). Gene set enrichment analysis further indicated enrichment of EMT, oxidative stress, and TNF–NFκB signaling pathways in MVI Endo (Figure 3B). Pseudotime trajectory analysis positioned MVI Endo at the early stage of the developmental trajectory (Figures 3C–E). SCENIC analysis revealed increased activity of transcription factors such as ELK4 in MVI Endo (Figure 3F). Metabolic profiling indicated that MVI Endo displayed relatively lower metabolic activity compared with other endothelial states (Figure 3G).

### Cell–cell communication analysis

**FIGURE 4:**
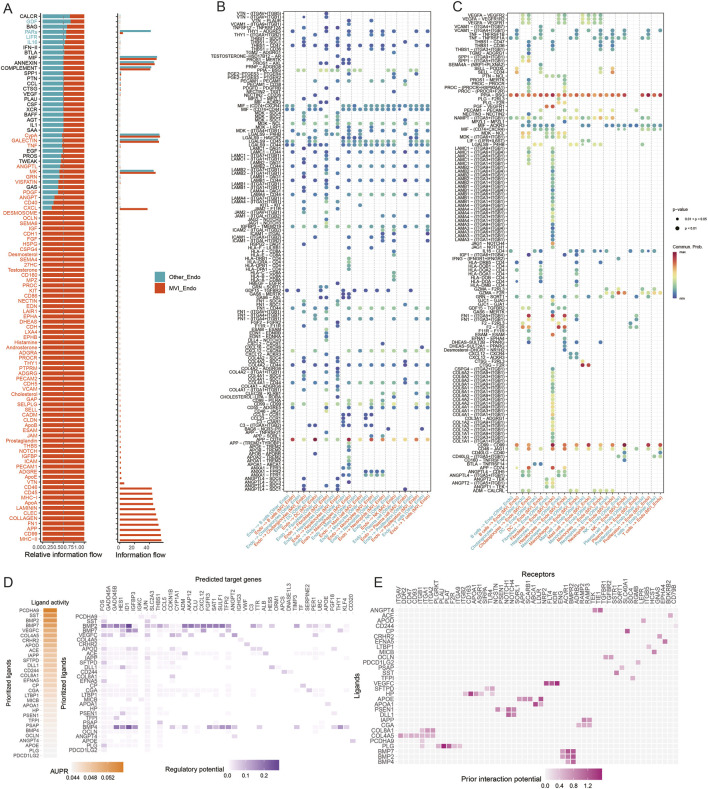
Cell–cell communication analysis. **(A)** Bar plot showing differences in intercellular communication; **(B)** dot plot of communication patterns with endothelial cells acting as signal senders; **(C)** dot plot of communication patterns with endothelial cells acting as signal receivers; **(D,E)** NicheNet-based analysis of differential cell–cell communication.

CellChat analysis revealed marked differences in intercellular communication between MVI Endo, other endothelial cells, and additional cell populations. MVI Endo showed enhanced activity of pro-angiogenic signaling pathways, including ANGPT and VEGF signaling (Figure 4A). Ligand–receptor analysis indicated that, when acting as signal senders, MVI Endo engaged malignant cells through ligand–receptor pairs associated with EMT and TGF-β signaling, and interacted with macrophages via BMP-related signaling linked to macrophage polarization (Figure 4B). When acting as signal receivers, strong TGF-β–related signaling was observed between fibroblasts and MVI Endo (Figure 4C), supporting a role for this endothelial subpopulation in EMT-associated processes. Subsequent NicheNet analysis further identified TGF-β and VEGF pathways as key mediators of communication between MVI Endo and malignant cells (Figures 4D,E).

### Spatial transcriptomic mapping

**FIGURE 5:**
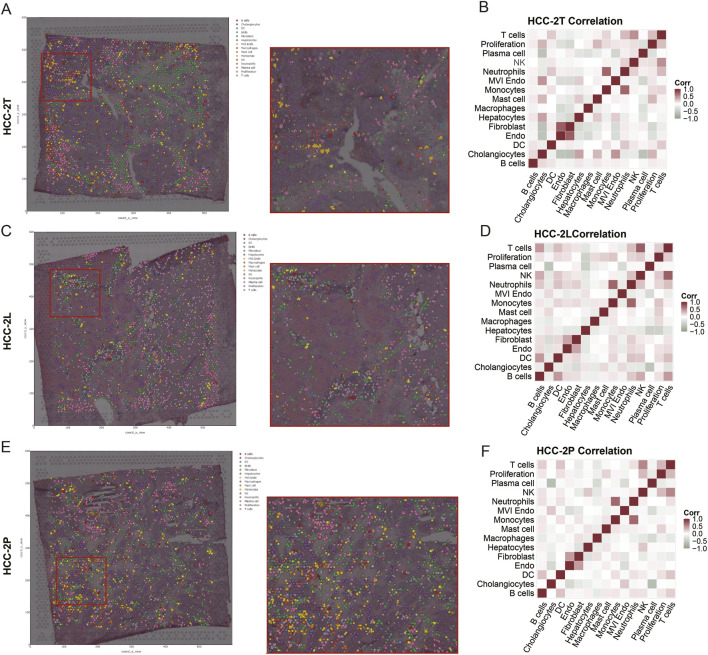
Spatial transcriptomic mapping. **(A)** CellTrek-based deconvolution mapping in HCC-2T; **(B)** CARD co-localization analysis in HCC-2T; **(C)** CellTrek-based deconvolution mapping in HCC-2L; **(D)** CARD co-localization analysis in HCC-2L; **(E)** CellTrek-based deconvolution mapping in HCC-2P; **(F)** CARD co-localization analysis in HCC-2P.

Spatial transcriptomic mapping was performed with CellTrek to further delineate cellular distributions in HCC-2T, HCC-2L, and HCC-2P sections. MVI Endo cells were consistently enriched in vascular regions adjacent to tumor-enriched areas, in line with their proposed biological functions. CARD-based spatial co-localization analysis further demonstrated significant spatial co-localization between MVI Endo cells and malignant cells (Figures 5A,B). Validation across additional sections showed a similar pattern, with MVI Endo cells preferentially localized around vascular regions and exhibiting spatial co-localization with tumor cells (Figures 5B,C).

### Construction of an MVI-Associated prognostic model using machine learning

**FIGURE 6:**
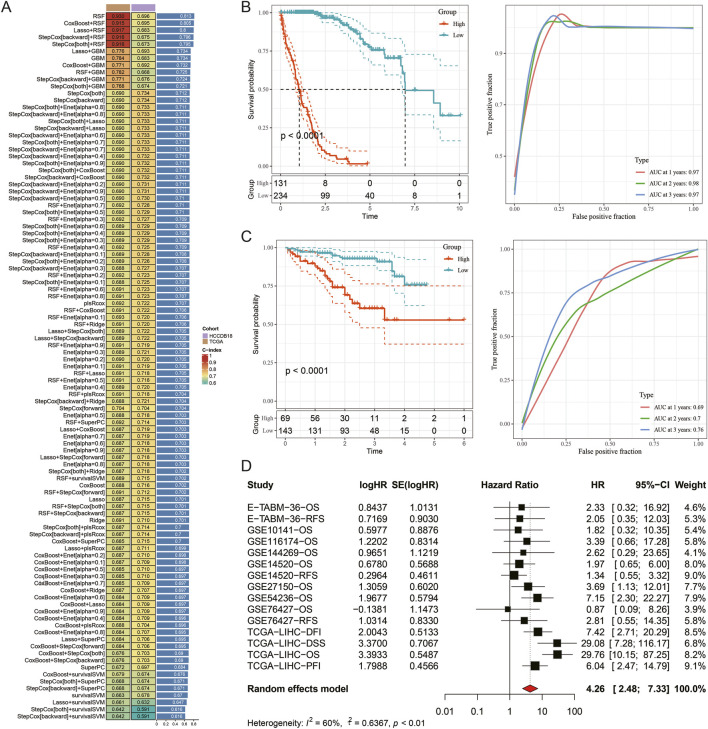
Construction of an MVI-associated prognostic model using machine learning. **(A)** Overview of 101 machine-learning model combinations; **(B)** Kaplan–Meier survival curves and 1-, 2-, and 3-year ROC analyses in the TCGA-LIHC cohort; **(C)** Kaplan–Meier survival curves and 1-, 2-, and 3-year ROC analyses in the HCCDB18 cohort; **(D)** Meta-analysis evaluating the prognostic performance of the model.

Based on key gene modules of MVI Endo, an integrative machine-learning framework was implemented to develop a consensus prognostic model. A total of 101 predictive models were fitted in the TCGA-LIHC and HCCDB18 cohorts, and the concordance index (C-index) was calculated for each model in the validation datasets. Considering both C-index performance and model complexity, the random survival forest (RSF) model was identified as the optimal approach (Figure 6A). In both training and validation cohorts, patients with higher risk scores consistently exhibited poorer survival outcomes (*p* < 0.05). In the TCGA-LIHC cohort, the 1-, 2-, and 3-year area under the ROC curves were 0.97, 0.98, and 0.97, respectively, whereas corresponding values in the HCCDB18 cohort were 0.69, 0.70, and 0.76 (Figures 6B,C). Meta-analysis across multiple datasets further confirmed that genes included in the model functioned as significant risk factors (*p* < 0.01) (Figure 6D).

### Immune infiltration analysis

**FIGURE 7:**
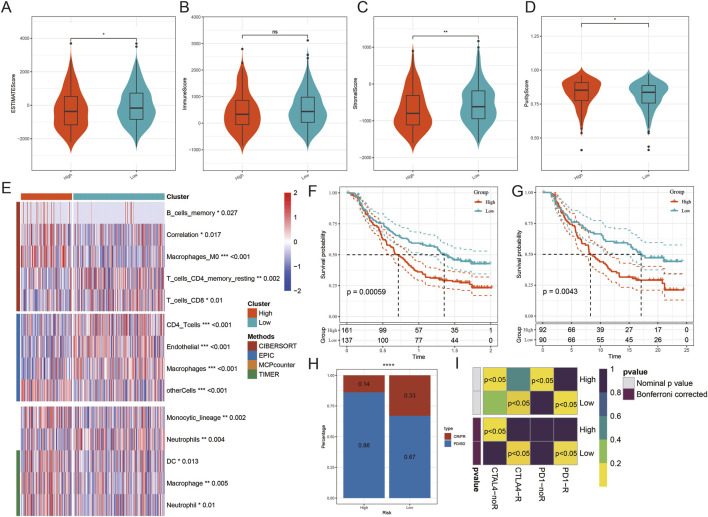
Immune infiltration analysis. **(A–D)** ESTIMATE score, immune score, stromal score, and tumor purity score in high- and low-risk groups; **(E)** immune cell infiltration differences between high- and low-risk groups; **(F)** overall survival in the IMvigor210 cohort; **(G)** overall survival in stage I–II patients from the IMvigor210 cohort; **(H)** proportions and differences of immunotherapy response between groups; **(I)** submap analysis.

ESTIMATE analysis showed that ESTIMATE and stromal scores were significantly higher in the high-risk group than in the low-risk group (*p* < 0.05), whereas tumor purity scores were also elevated in the high-risk group (*p* < 0.001) (Figures 7A–D). Subsequent immune infiltration analysis indicated a marked reduction of immune cytotoxic cell populations in the high-risk group (Figure 7E). Model-based deconvolution in the IMvigor210 cohort revealed that patients with higher risk scores experienced worse overall survival (Figure 7F). After stratification by clinical stage, patients with stage I–II disease in the high-risk group likewise exhibited poorer prognosis (Figure 7G). In addition, patients in the high-risk group showed reduced responsiveness to atezolizumab, with significantly increased proportions of progressive disease and stable disease (PD/SD) (Figure 7H). Consistently, submap analysis indicated diminished predicted benefit from immunotherapy in the high-risk group (Figure 7I).

### Single-gene spatial transcriptomic analysis

**FIGURE 8:**
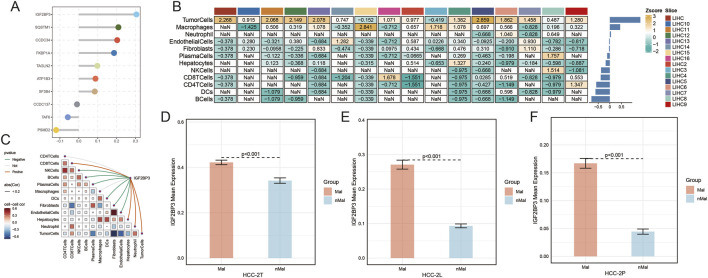
Single-gene spatial transcriptomic analysis. **(A)** Multivariate Cox regression analysis of model genes; **(B,C)** heatmap of correlations between IGF2BP3 and cell types in spatial transcriptomic data; **(D–F)** bar plots showing differential distribution of IGF2BP3 in tumor regions of HCC-2T, HCC-2L, and HCC-2P sections.

Multivariate Cox regression analysis of model genes identified IGF2BP3 as the most influential prognostic factor (Figure 8A). Correlation analysis based on spatial transcriptomic data from LIHC samples demonstrated that IGF2BP3 expression was positively associated with both tumor cells and endothelial cells (Figures 8B,C). Spatial distribution analysis further showed that IGF2BP3 was consistently and significantly upregulated in tumor regions across all three sections (Figures 8D–F).

### IGF2BP3 promotes proliferation and tube formation of tumor-associated endothelial cells

**FIGURE 9:**
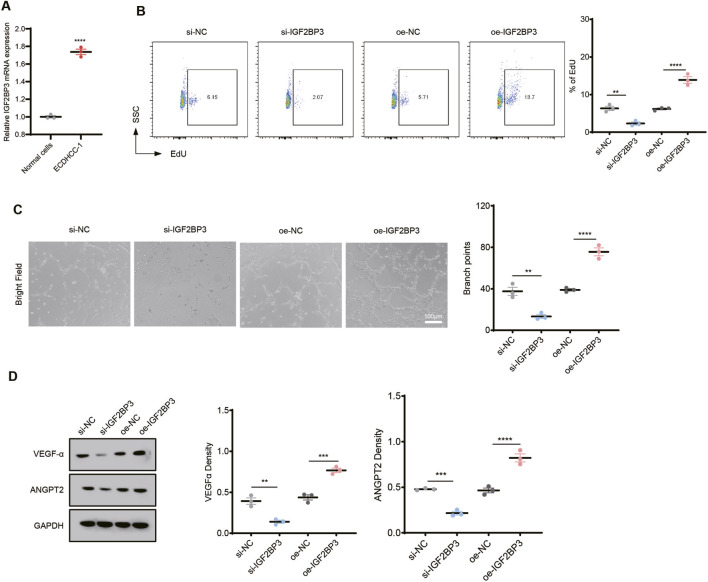
IGF2BP3 promotes proliferation and angiogenesis of tumor-associated endothelial cells. **(A)** qRT-PCR analysis of IGF2BP3 expression in normal endothelial cells (HUVECs) and hepatocellular carcinoma–derived endothelial cells (ECDHCC-1), showing significantly higher expression in ECDHCC-1 cells (*p* < 0.001); **(B)** EdU staining assessing the effect of IGF2BP3 on ECDHCC-1 cell proliferation, with reduced proliferation upon IGF2BP3 silencing and enhanced proliferation upon IGF2BP3 overexpression (*p* < 0.05); **(C)** tube formation assay showing decreased tube-forming capacity in the si-IGF2BP3 group and increased branch formation in the oe-IGF2BP3 group (*p* < 0.01); **(D)** Western blot analysis of angiogenesis-related proteins VEGF-A and ANGPT2, showing downregulation upon IGF2BP3 silencing and upregulation upon IGF2BP3 overexpression (*p* < 0.001). Data are presented as mean ± SD, and statistical significance was assessed with student’s t-test.

To assess the regulatory role of IGF2BP3 in tumor-associated endothelial cells, qRT-PCR was first performed to measure IGF2BP3 expression in normal endothelial cells (HUVECs) and hepatocellular carcinoma–derived endothelial cells (ECDHCC-1). IGF2BP3 expression was significantly higher in ECDHCC-1 cells (*p* < 0.001) (Figure 9A), suggesting a potential involvement in tumor endothelial cell function. Stable ECDHCC-1 cell lines with IGF2BP3 knockdown (si-IGF2BP3) or overexpression (oe-IGF2BP3) were subsequently established, and cell proliferation was evaluated with the EdU assay. Compared with control groups, IGF2BP3 silencing markedly suppressed ECDHCC-1 cell proliferation, whereas IGF2BP3 overexpression significantly enhanced proliferative activity (*p* < 0.05) (Figure 9B). Consistent with these findings, tube formation assays showed a significant reduction in capillary-like structures in the si-IGF2BP3 group and a pronounced increase in the oe-IGF2BP3 group, indicating that IGF2BP3 enhances endothelial angiogenic capacity (*p* < 0.01) (Figure 9C). Western blot analysis further confirmed that expression levels of the angiogenesis-related proteins VEGF-A and ANGPT2 were significantly decreased upon IGF2BP3 silencing and increased upon IGF2BP3 overexpression (*p* < 0.001) (Figure 9D). Collectively, these results indicate that IGF2BP3 is highly expressed in tumor-associated endothelial cells and promotes endothelial proliferation and vascular structure formation, thereby reinforcing a pro-angiogenic phenotype that supports tumor vascularization.

## Discussion

In this work, we identified an endothelial cell subpopulation associated with microvascular invasion (MVI Endo) that shows a distinct transcriptional profile together with increased activity of pro-angiogenic programs, epithelial–mesenchymal transition (EMT), and TGF-β–responsive signaling. By integrating gene programs derived from GeneNMF with co-expression modules defined by hdWGCNA, we extracted a core gene module with stable expression characteristics that was strongly associated with unfavorable clinical outcomes. Spatial transcriptomic analyses further showed that MVI Endo cells preferentially localize at the tumor–vascular interface, suggesting a functional involvement in the formation of microvascular invasion. These observations emphasize the contribution of endothelial heterogeneity to tumor progression and indicate that therapeutic strategies targeting endothelial states, rather than single molecular targets, may be more effective in modulating tumor angiogenesis.

At the mechanistic level, our data suggest that MVI Endo cells reside within an activated microvascular niche enriched for pro-invasive signaling pathways, including TGF-β, VEGF/ANGPT2, and NF-κB, and participate in extensive ligand–receptor interactions with fibroblasts and tumor cells. Such signaling contexts have been linked in prior studies to endothelial–mesenchymal transition (EndMT), extracellular matrix remodeling, and increased vascular permeability, processes that facilitate tumor cell transmigration across the vascular barrier. Consistent with this notion, pseudotime analysis positioned MVI Endo at an early stage of the endothelial developmental trajectory, indicating that this subpopulation may represent an invasion-permissive endothelial state shaped by early reprogramming events. Taken together, these findings support the view that MVI arises from a dynamic and cooperative process involving reciprocal interactions between endothelial and tumor cells.

The pronounced activation of pro-angiogenic and TGF-β signaling observed in MVI Endo points to the potential clinical relevance of precisely modulating the vascular microenvironment. Increasing evidence indicates that epigenetic regulation, particularly RNA m^6^A modification and DNA methylation, plays an important role in tumor angiogenesis and treatment response. IGF2BP3, a representative m^6^A reader, has been reported to stabilize m^6^A-modified transcripts such as NRF2, thereby enhancing antioxidant capacity and contributing to sorafenib resistance in hepatocellular carcinoma (Aibar et al., 2017). In addition, IGF2BP3 has been implicated in the stabilization of transcripts encoding pro-angiogenic factors, including VEGFA and ANGPT2, which directly promotes endothelial proliferation, tube formation, and vascular permeability, creating a vascular niche favorable for microvascular invasion (Liu et al., 2022). At the post-transcriptional level, m^6^A modification governs the stability of key angiogenic transcripts, providing a rapid epigenetic mechanism for vascular remodeling. Clinically, our data imply that IGF2BP3 functions as an upstream stabilizer of the VEGF/ANGPT2 and TGF-β signaling networks, rather than acting in isolation. Consequently, we propose a hierarchical therapeutic strategy: targeting the IGF2BP3/m^6^A axis to destabilize the mRNA of pro-invasive factors could sensitize Tumor-Associated Endothelial Cells (TAECs) to standard anti-angiogenic or TGF-β inhibitors. This combinatorial approach offers a mechanistic rationale for overcoming the vascular plasticity and therapeutic resistance frequently observed in MVI-positive HCC, moving beyond simple additive effects to address the root causes of vascular remodeling.

By systematically integrating single-cell and spatial multi-omics data, this study constructs a comprehensive endothelial atlas of microvascular invasion in hepatocellular carcinoma, identifies an MVI Endo subpopulation with pro-angiogenic, EMT-like, and TGF-β–responsive features, and establishes a high-performance prognostic model based on its core gene module using 101 algorithmic combinations. Functional experiments further demonstrate that IGF2BP3 enhances proliferation and tube formation in tumor-associated endothelial cells, supporting its role in MVI-associated vascular remodeling.

Spite the novel insights provided by this study, we acknowledge certain limitations. Notably, the single-cell RNA sequencing dataset utilized for the initial discovery consists of a relatively small cohort (three MVI-positive and two MVI-negative samples). Although we applied rigorous quality control and integration algorithms (e.g., Harmony and GeneNMF) to minimize batch effects and identify robust gene programs, the limited sample size may not fully capture the complete spectrum of inter-patient heterogeneity associated with MVI. To address this, we integrated spatial transcriptomics to validate the spatial distribution of the identified endothelial subpopulation and employed multiple large-scale external bulk RNA-seq cohorts (e.g., TCGA-LIHC, HCCDB18) to confirm the clinical relevance and prognostic value of the MVI-associated signature. Furthermore, *in vitro* experiments were conducted to verify the functional role of the core driver gene, IGF2BP3. Future studies incorporating larger single-cell cohorts are warranted to further refine these findings and explore specific variations across broader patient populations.

## Conclusion

In summary, this study identifies a key endothelial cell subpopulation driving microvascular invasion in hepatocellular carcinoma and outlines its regulatory framework, establishes the central role of IGF2BP3 in tumor-associated endothelial remodeling, and proposes translatable vascular-targeted and combinatorial therapeutic strategies that may contribute to improving the poor prognosis associated with MVI.

## Data Availability

The original contributions presented in the study are included in the article/supplementary material, further inquiries can be directed to the corresponding author.
